# Multicentre patterns-of-care analysis of adjuvant and definitive (chemo)radiotherapy for pharyngeal and laryngeal carcinoma in Franconia, Germany

**DOI:** 10.1007/s00066-026-02550-z

**Published:** 2026-05-11

**Authors:** Karoline Pollmann, Adriana Salazar Hammann, Jochen Willner, Reinhart Sweeney, Stefan Münch, Thomas Gehrke, Michael Flentje, Andrea Wittig-Sauerwein, Victor Lewitzki

**Affiliations:** 1 Department of Radiotherapy and Radiation Oncology, University Hospital Würzburg, Josef-Schneider-Str. 11, 97080 Würzburg, Germany; 2 Department of Radiation Oncology, Sozialstiftung Bamberg, Bamberg, Germany; 3 Department of Radiation Oncology, Klinikum Bayreuth, Bayreuth, Germany; 4 Department of Radiation Oncology, Leopoldina Hospital Schweinfurt, Schweinfurt, Germany; 5 Department of Oto-Rhino-Laryngology, Plastic, Aesthetic and Reconstructive Head and Neck Surgery, University of Würzburg, Würzburg, Germany

**Keywords:** Head and neck squamous cell carcinoma (HNSCC), Radiotherapy, Tertiary versus secondary hospitals, HPV status, Survival analysis (OS/PFS/LRRFS)

## Abstract

**Purpose:**

To compare treatment patterns and oncologic outcomes of patients with pharyngeal and laryngeal squamous cell carcinoma treated at a tertiary hospital versus three secondary regional hospitals in Franconia, Germany.

**Methods:**

We retrospectively analyzed 308 consecutive patients (tertiary hospital *n* = 164; secondary regional hospitals *n* = 144) diagnosed between 01/2017 and 12/2019 and treated with primary or adjuvant radiotherapy (RT), with or without concomitant chemotherapy. Primary endpoints were overall survival (OS), progression-free survival (PFS), and locoregional recurrence-free survival (LRRFS). Kaplan-Meier estimates, multivariable Cox regression models, and a 1:1 matched-pair analysis were applied. Residual confounding was quantified using E‑values.

**Results:**

The cohorts were well balanced, except for a higher prevalence of HPV-negative tumors (52% vs 42%, *p* = 0.004) and ECOG performance status > 1 (20% vs 9%, *p* = 0.004) in secondary hospitals. Median follow-up was 34 months. OS (median 35.5 vs 32 months; HR 0.90, 95% CI 0.72–1.12) and PFS (29 vs 21.5 months; HR 0.86, 95% CI 0.69–1.07) did not differ significantly between groups. LRRFS was shorter in secondary hospitals (32 vs 25.5 months; HR 1.25, 95% CI 1.06–1.48; *p* = 0.05), which may partly be explained by the higher rate of HPV-negative tumors in this cohort. Sensitivity analyses indicated that residual confounding may still have contributed to this difference. Multivariable analysis identified ECOG > 1 (HR 3.29), HPV negativity (HR 2.56), R2 resection status (HR 16.1 for PFS), and a cumulative cisplatin dose < 160 mg/m^2^ (HR > 3) as independent prognostic factors.

**Conclusion:**

Radiotherapy for pharyngeal and laryngeal cancer achieved comparable OS and PFS in tertiary and secondary hospitals that adhere to current quality benchmarks. The observed difference in locoregional control, which was limited to the primary (non-surgical) cohort, is consistent with the higher proportion of HPV-negative and functionally impaired patients treated in secondary hospitals. Residual imbalance after adjustment and matching indicates that unmeasured clinical or treatment-related factors may also contribute. Prompt initiation of RT and adequate cumulative cisplatin dosing remain critical.

## Introduction

Head and neck squamous cell carcinoma (HNSCC) of the pharynx and larynx remains a major global health burden, with approximately 900,000 new cases and more than 450,000 deaths annually [1, 2]. Despite advances in multimodality treatment, population-based overall survival (OS) remains at around 60% [2]. Guideline-concordant management requires multidisciplinary decision-making, risk-adapted radiotherapy (RT), and, where indicated, surgery [3, 4].

The pivotal postoperative trials EORTC 22931 and RTOG 9501 demonstrated the benefit of adding high-dose cisplatin to adjuvant RT in patients with positive margins or extracapsular nodal extension [5, 6], and a recently published combined analysis provided updated long-term insights into these cohorts [7]. Successive MACH-NC meta-analyses confirmed the benefit of concomitant chemotherapy in both postoperative and definitive settings [1, 2, 8].

Beyond tumor biology, institutional factors such as center volume, guideline adherence, and radiotherapy quality assurance (QA) have been associated with patient outcomes. The National Comprehensive Cancer Network (NCCN) therefore recommends treatment at high-volume, specialized centers [4, 9–11]. Wuthrick et al. [12] showed a 15% relative reduction in mortality for patients enrolled in cooperative-group trials at high-accrual institutions.

Nevertheless, decentralized care structures are essential to ensure timely access to treatment, particularly in geographically widespread regions with limited transportation infrastructure. We studied an area of Northern Bavaria, served by a tertiary academic radiotherapy center and three secondary hospitals equipped with intensity-modulated radiotherapy (IMRT) and volumetric modulated arc therapy (VMAT). Whether oncologic outcomes differ between these care settings in the contemporary IMRT era remains insufficiently elucidated.

We retrospectively analyzed 308 consecutive patients with newly diagnosed oropharyngeal, hypopharyngeal, or laryngeal squamous cell carcinoma treated with curative-intent radiotherapy or chemoradiotherapy between 2017 and 2019 at the tertiary center and the three secondary hospitals. We selected OS, PFS, and LRRFS as primary quality indicators, as these endpoints reflect both overall mortality and locoregional tumor control and are widely used as benchmark parameters in head and neck radiotherapy trials [13, 14].

## Patients and methods

### Study design and oversight

This retrospective multicenter cohort study compared curative-intent radiotherapy or chemoradiotherapy delivered at a tertiary academic radiotherapy center and three secondary hospitals. The study design and reporting followed STROBE recommendations for observational research [15]. The study was approved by the local ethics committee (file 2020123) and conducted according to the Declaration of Helsinki.

### Eligibility

**Fig. 1:**
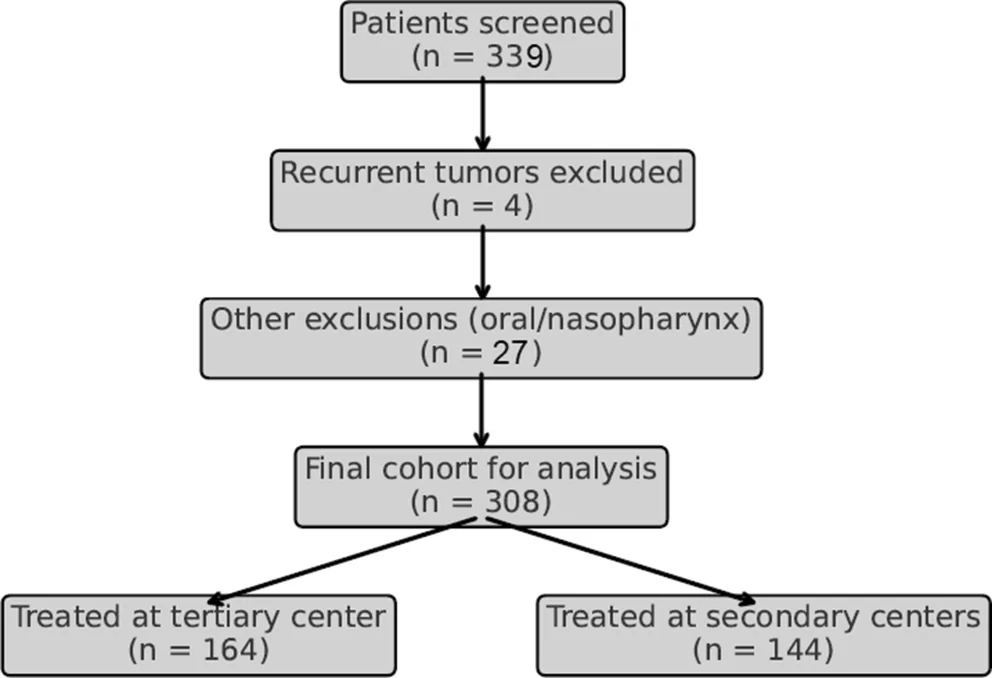
CONSORT flow diagram of patient selection. A total of 339 patients were screened; patients with recurrent tumors or other tumor entities were excluded.

Inclusion criteria were: (i) histologically confirmed SCC of the oropharynx, hypopharynx, or larynx (ICD-10 C01, C05, C09–C13, C32; overlaps C14/C39); (ii) curative-intent external-beam RT (definitive ≥ 66 Gy or postoperative ≥ 60 Gy); and (iii) complete electronic records. Patients with prior head and neck RT, distant metastases, palliative treatment intent, incomplete RT (< 95% of prescribed dose), or missing follow-up were excluded. After screening 339 cases, 308 fulfilled all inclusion criteria (164 cases treated in the tertiary center; 144 cases treated in the secondary hospitals), as shown in Fig. 1.

### Data collection

Demographic, tumor, and treatment data were extracted from radiotherapy information systems, hospital information systems, and tumor documentation systems of the respective hospitals (SIS, MOSAIQ, SAP), as well as from the regional cancer registry of the University Hospital (ONKOSTAR software). Variables included age, sex, smoking status, ECOG performance status, UICC7 stage, HPV status (p16 IHC or DNA PCR), and resection status (R0–R2). Diagnostic work-up was largely comparable between the participating centers and followed established institutional standards and national guidelines. This included cross-sectional imaging in all patients. However, the use of specific modalities such as MRI or PET/CT was not fully standardized across centers and was applied based on clinical indication and local practice. Treatment parameters included treatment intent (definitive vs adjuvant), radiotherapy fractionation, total and per-fraction dose, planning target volume (PTV), systemic therapy (agent, schedule, cumulative dose), and three time intervals: TTI (time interval between diagnosis and RT), SRT (time interval between surgery and RT), TPT (time interval between surgery and end of RT). Median values by hospital type are presented in Table 1.

**Table 1:** Treatment-related time intervals and radiotherapy interruptions. Shown are median time to treatment initiation (TTI; diagnosis to start of radiotherapy), interval from surgery to radiotherapy (SRT), and time from surgery to completion of radiotherapy (TPT), as well as metrics related to radiotherapy interruptions and treatment discontinuation. Comparisons are shown for tertiary versus secondary hospitals.

Interval/Category	Total (*N* = 308)	Tertiary hospital (*n* = 164)	Secondary hospitals (*n* = 144)
TTI ≤ 6 weeks, *n* (%)	263 (85.4%)	153 (93.3%)	110 (76.4%)
Median TTI (weeks), (range)	4 (0–45)	3 (0–21)	4 (0–45)
Median SRT (weeks)†, (range) †	5 (2–14)	4 (2–12)	6 (4–14)
Median TPT (weeks)†, (range) †	11 (5–21)	10 (5–21)	13 (5–21)
Any RT interruption (≥ 1 day)	17 (5.5%)	6 (3.7%)	11 (7.6%)
RT interruption > 5 days	3 (1.0%)	2 (1.2%)	1 (0.7%)
RT discontinued (< 30 fractions)	24 (7.8%)	11 (6.7%)	13 (9.0%)

Abbreviations: *TTI* time to treatment initiation; *SRT* surgery to radiotherapy time; *TPT* treatment package time; *RT* radiotherapy; *PTV* planning target volume

† Operated patients only (*n* = 151)

### Institutional characteristics and quality assurance

Institutional quality assurance parameters, including hospital and departmental certifications, were systematically assessed for all participating clinics and are summarized in Table 2. Only the tertiary hospital is certified as a dedicated head and neck cancer center. QA parameters were not used for stratification or outcome adjustment. Other potential structural or process parameters (e.g., tumor boards, staffing levels) could not be assessed due to lack of standardized data collection.

**Table 2:** Institutional quality assurance characteristics of participating hospital types. Displayed are hospital certification and structural quality indicators for the tertiary academic center and secondary regional hospitals. Parameters include certified hospital quality management system (DIN EN ISO 9001), certified ENT department, and certification as a dedicated head and neck cancer center. Quality assurance parameters were assessed descriptively and were not used for stratification or outcome adjustment.

Parameter	Tertiary hospital (*n* = 164)	Secondary hospitals (*n* = 144)
Certified hospital quality management system	DIN EN ISO 9001	Partially DIN EN ISO 9001
Certified ENT department	Yes	No
Certified head and neck cancer center	Yes	No

Abbreviations: *ENT* Ear, Nose, and Throat

### Endpoints

Primary endpoints were OS, PFS, and LRRFS. OS was measured from the date of initial diagnosis to death from any cause, whereas PFS and LRRFS were measured from the first radiotherapy fraction to disease progression or locoregional relapse, respectively. Secondary endpoints were treatment interruptions (≥ 1 missed fraction), RT prolongation (> 5 days vs planned), cumulative cisplatin dose, and RT quality assurance metrics.

### Radiotherapy and systemic treatment

All clinics used CT-based IMRT/VMAT with daily image guidance. Target delineation followed DAHANCA and Grégoire et al. consensus atlases [16]. Prescription dose at the tertiary center was typically 69.3 Gy in 33 fractions for definitive treatments and 66 Gy in 30 fractions in adjuvant settings, where simultaneous integrated boost (SIB) was used in all cases with 1–2 sequential boost fractions in all but 5 patients. Secondary hospitals used comparable prescriptions but more regularly utilized a sequential boost after the SIB for the first series. The prescribed dose to the elective nodal irradiation (ENI) volume was identical (66 Gy) across all centers, with minor deviations due to the different prescriptions in SIB and sequential boost protocols. Systemic therapy followed German guidelines, recommending cisplatin 100 mg/m^2^ q3w or weekly 40 mg/m^2^.

### Statistical analysis

Continuous variables were compared with Student’s t‑test or ANOVA; categorical variables with χ^2^ or Fisher’s exact test. Survival curves were generated with Kaplan-Meier estimates and compared using the log-rank test. Multivariable Cox proportional hazards models adjusted for ECOG, HPV, resection status, cisplatin dose, treatment intent, and RT completion. Proportional hazards (PH) assumptions were verified via Schoenfeld residuals [17].

### Matching algorithm

To minimize selection bias, a 1:1 optimal pair match was performed using the MatchIt package (R v4.4.2) to balance covariates: age, sex, smoking, UICC stage, HPV, perineural invasion, resection status, treatment intent, and chemotherapy. Balance was confirmed by a standardized mean difference < 0.10.

### Sensitivity analyses

Post-hoc power was estimated with Freedman’s formula [19]. Sensitivity analysis using the E‑value followed the method of VanderWeele and Ding [18]. All analyses were performed with SPSS v28 and R v4.4.2, using two-sided α = 0.05.

## Results

### Patient characteristics

A total of 308 patients were eligible (164 treated at the tertiary academic center, 144 at secondary regional hospitals). The distribution of patients in the secondary hospitals was as follows: 59 patients in Secondary Hospital A, 45 in Secondary Hospital B, and 40 in Secondary Hospital C. Baseline demographic and tumor characteristics are summarized in Table 3. Groups were well balanced except for a higher prevalence of HPV-negative tumors (52% vs 42%, *p* = 0.004) and more patients with ECOG > 1 (20% vs 9%, *p* = 0.004) in secondary regional hospitals. Given the relatively small patient numbers in each secondary hospital, these clinics were analyzed as a combined cohort to provide sufficient statistical power for outcome analyses, while maintaining comparability with the tertiary center.

**Table 3:** Baseline patient characteristics. Variables include age, sex, ECOG performance status, UICC stage, HPV status, and resection status. Secondary hospitals were analyzed as a combined cohort.

Parameter	Total (*n* = 308)	Tertiary hospital (*n* = 164)	Secondary hospitals (*n* = 144)	*p*-Value
Age at diagnosis (years), median (range)/mean ± SD	62 (31–90)/63.4 ± 9.9	64 (31–85)/64.5 ± 9.6	61 (33–90)/62.3 ± 10.2	0.057
Sex—male	245 (79.5%)	132 (80.5%)	113 (78.5%)	0.662
ECOG 0–1	256 (83.1%)	147 (89.6%)	109 (75.7%)	< 0.001
HPV-negative	144 (46.8%)	69 (42.1%)	75 (52.1%)	0.004
HPV-positive	78 (25.3%)	53 (32.3%)	25 (17.4%)	–
HPV unknown/not tested	86 (27.9%)	42 (25.6%)	44 (30.6%)	–
UICC stage III–IV	270 (87.7%)	142 (86.6%)	128 (88.9%)	n. s.
T stage T1–2	119 (38.6%)	55 (33.5%)	64 (44.4%)	n. s.
T stage T3–4	188 (61.0%)	109 (66.0%)	79 (54.9%)	n. s.
N stage N0	63 (20.5%)	36 (22.0%)	27 (18.8%)	n. s.
N stage N1	59 (19.2%)	32 (19.5%)	27 (18.8%)	n. s.
N stage N2	147 (47.7%)	72 (43.9%)	75 (52.1%)	n. s.
N stage N3	38 (12.3%)	24 (14.6%)	14 (9.7%)	n. s.
N stage NX	1 (0.3%)	0 (0.0%)	1 (0.7%)	n. s.
Resection R0	101 (32.8%)	53 (32.3%)	48 (33.3%)	n. s.
Resection R1	26 (8.4%)	19 (11.6%)	7 (4.9%)	n. s.
Resection R2	5 (1.6%)	3 (1.8%)	2 (1.4%)	n. s.
Resection RX	14 (4.5%)	9 (5.5%)	5 (3.5%)	n. s.
No previous surgery	157 (51.0%)	80 (48.8%)	77 (53.5%)	n. s.

### Treatment delivery

Details of radiotherapy and systemic therapy are shown in Table 4. Treatment intent (definitive vs adjuvant) was balanced (*p* = 0.36). In operated cases, surgery was predominantly performed at dedicated head and neck cancer centers, accounting for 100% of cases at the tertiary center and 72.4% of cases referred from the regional hospitals. Median total prescribed RT dose and fractionation schedules were comparable between groups. The cumulative cisplatin dose did not differ significantly between hospital types, with secondary hospitals having a slightly higher proportion of patients receiving < 160 mg/m^2^ (22.1% vs 17.5%).

**Table 4:** Treatment characteristics. Shown are treatment intent (definitive vs adjuvant), prescribed radiotherapy dose and fractionation, detailed fractionation techniques (SIB vs. sequential boost), mean applied doses, and cumulative cisplatin dose categories. Data are presented for tertiary versus secondary hospitals.

Variable	Total	Tertiary hospital	Secondary hospitals	*p*-value
*Primary RT*	157 (51.0%)	80 (48.8%)	77 (53.5%)	–
*Adjuvant RT*	151 (49.0%)	84 (51.2%)	67 (46.5%)	–
*Surgery at specialized HNC centers*	–	100%	72.4%	–
*Surgery at regional hospitals*	–	0%	23.4%	–
*Median prescribed total dose [Gy] in PTV1 median (range)*	68.0 (54–73)	69.1 (60–73)	68.0 (54–73)	–
*Median number of fractions*	33	30	33	–
*Cisplatin cumulative dose ≥* *200* *mg/m*^2^*	76/140 (54.3%)	31/77 (40.3%)	45/63 (71.4%)	–
*Cisplatin cumulative dose 160–199* *mg/m*^2^*	36/140 (25.7%)	29/77 (37.7%)	7/63 (11.1%)	–
*Cisplatin cumulative dose <* *160* *mg/m*^2^*	28/140 (20.0%)	17/77 (22.1%)	11/63 (17.5%)	–
Radiation technique (Primary RT)*	*n* = 150	*n* = 77	*n* = 73	< 0.001 ***
SIB only	95 (63.3%)	63 (81.8%)	32 (43.8%)	–
SIB with sequential boost	29 (19.3%)	2 (2.6%)	27 (37.0%)	–
3D-Technique	1 (0.7%)	0 (0%)	1 (1.4%)	–
Mixed/Unclear	3 (2.0%)	0 (0%)	3 (4.1%)	–
Doses and volumes (Primary RT)**	*n* = 131	*n* = 65	*n* = 66	–
Mean Dose Elective [Gy]	–	64.2 (± 1.8)	58.2 (± 4.6)	< 0.001 ***
Mean Dose Boost 1 [Gy]	–	68.7 (± 1.3)	64.3 (± 3.6)	< 0.001 ***
Mean Dose Boost 2 [Gy]	–	70.9 (± 1.1)	68.9 (± 3.4)	< 0.001 ***
Volume Elective [ccm]	–	972.2 (± 321.2)	1176.3 (± 427.3)	0.003 **
Volume Boost 1 [ccm]	–	432.7 (± 217.5)	604.6 (± 366.5)	0.003 **
Volume Boost 2 [ccm]	–	85.9 (± 61.7)	212.3 (± 176.5)	< 0.001 ***
Radiation technique (Adjuvant RT)*	*n* = 147	*n* = 83	*n* = 64	< 0.001 ***
SIB only	94 (63.9%)	75 (90.4%)	19 (29.7%)	–
SIB with sequential boost	36 (24.5%)	3 (3.6%)	33 (51.6%)	–
3D-Technique	3 (2.0%)	0 (0%)	3 (4.7%)	–
Mixed/Unclear	1 (0.7%)	0 (0%)	1 (1.6%)	–
Doses and volumes (Adjuvant RT)**	*n* = 135	*n* = 78	*n* = 57	–
Mean Dose Elective [Gy]	–	62.6 (± 2.5)	55.4 (± 3.2)	< 0.001 ***
Mean Dose Boost 1 [Gy]	–	65.9 (± 2.0)	61.3 (± 2.6)	< 0.001 ***
Mean Dose Boost 2 [Gy]	–	67.9 (± 1.7)	64.5 (± 4.1)	< 0.001 ***
Volume Elective [ccm]	–	854.1 (± 325.1)	1076.8 (± 428.1)	0.001 **
Volume Boost 1 [ccm]	–	445.0 (± 230.1)	502.9 (± 262.7)	0.335
Volume Boost 2 [ccm]	–	268.6 (± 183.7)	181.9 (± 118.6)	0.001 **

* Administered cisplatin chemotherapy only (*n* = 140). Dosimetric parameters (mean applied doses, PTV volumes, and technique distributions) are based exclusively on patients who completed the full prescribed curative radiotherapy course without premature discontinuation.

**Fig. 2:**
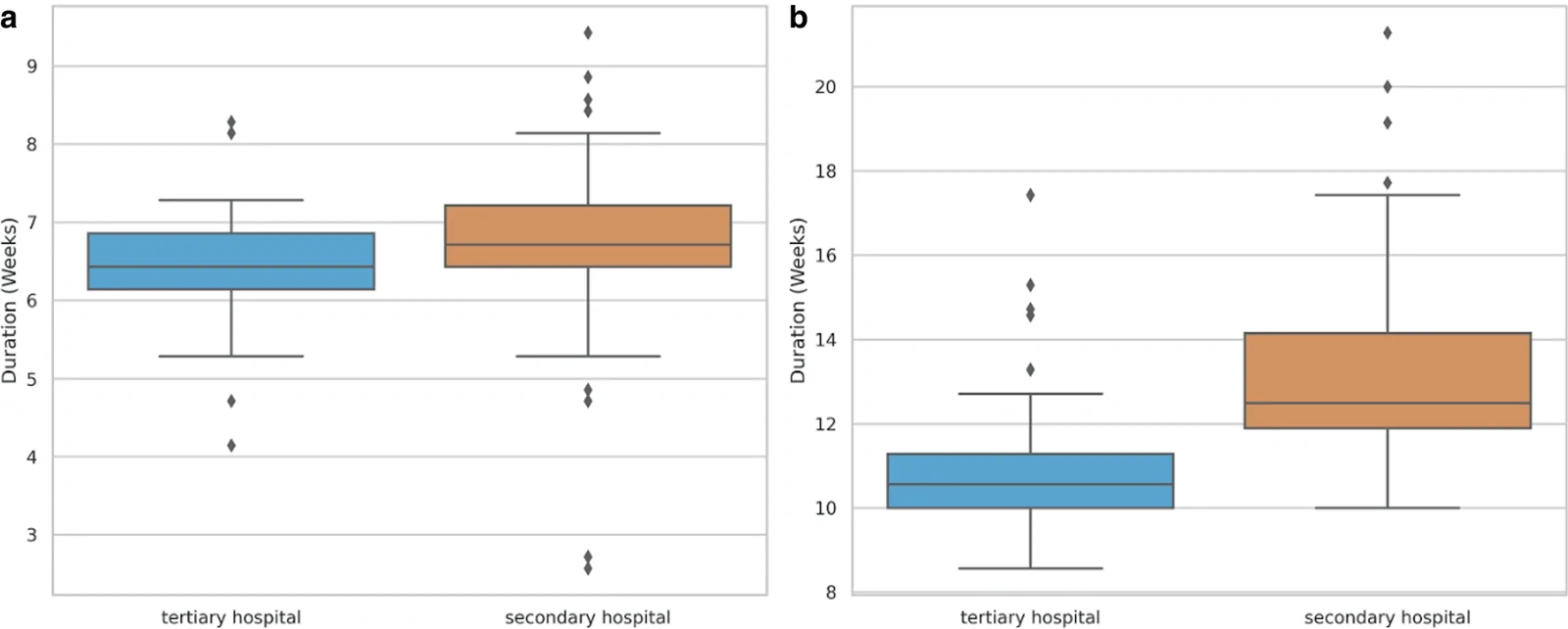
Treatment timelines (weeks). Boxplots comparing tertiary and secondary hospital types for (**a**) radiotherapy duration (completed cases) and (**b**) total time from surgery to radiotherapy completion (adjuvant cases). Whiskers represent 1.5 × IQR

Treatment-related time intervals are presented in Table 1. TTI was significantly shorter in the tertiary center (median 3 vs 4 weeks, *p* < 0.001). RT discontinuation before the planned 30 fractions in the curative setting occurred in 7.8% (*n* = 24) of the entire cohort (tertiary 6.7% vs secondary 9.0%). Unplanned RT interruptions (≥ 1 day) were low in 5.5% (*n* = 17) overall, and interruptions exceeding 5 consecutive days were rare (1.0%, *n* = 3). In all metrics pertaining to treatment breaks, no significant differences between tertiary and secondary clinic type were observed (Table 1). Overall treatment duration was significantly shorter for definitive treatment subgroup—mean of 47.1 vs 49.6 days (*p* = 0.0011) and adjuvant therapy subgroup with completed treatment mean of 43.9 vs 44.9 days (*p* = 0.0009) in tertiary vs secondary hospitals (Table 5). In adjuvant cases, the median interval from surgery to radiotherapy completion was significantly shorter at the tertiary hospital compared to the secondary hospitals (73.5 vs 89.0 days; *p* < 0.001) (Fig. 2).

**Table 5:** Treatment durations (in days) by hospital type. Data presented as *N*, mean ± SD, and median. Subgroups compare tertiary vs. secondary hospitals across all patients, completed treatments and adjuvant modalities. *P*-values determined by Mann-Whitney U test.

Subgroup	Hospital	*N*	Mean	Median	SD	*p*-value (MWU)
Overall treatment duration (All Patients)	Tertiary	164	43.76	45.0	7.45	*0.0014*
	Secondary	144	45.27	46.0	9.16	–
Completed Treatment	Tertiary	147	45.41	46.0	3.88	*<* *0.0001*
	Secondary	126	47.44	47.0	6.15	–
Primary RCT (Completed)	Tertiary	68	47.12	47.0	4.05	*0.0011*
	Secondary	67	49.60	49.0	6.41	–
Adjuvant RCT (Completed)	Tertiary	79	43.95	44.0	3.06	*0.0009*
	Secondary	59	44.98	45.0	4.83	–

### Survival outcomes

**Fig. 3:**
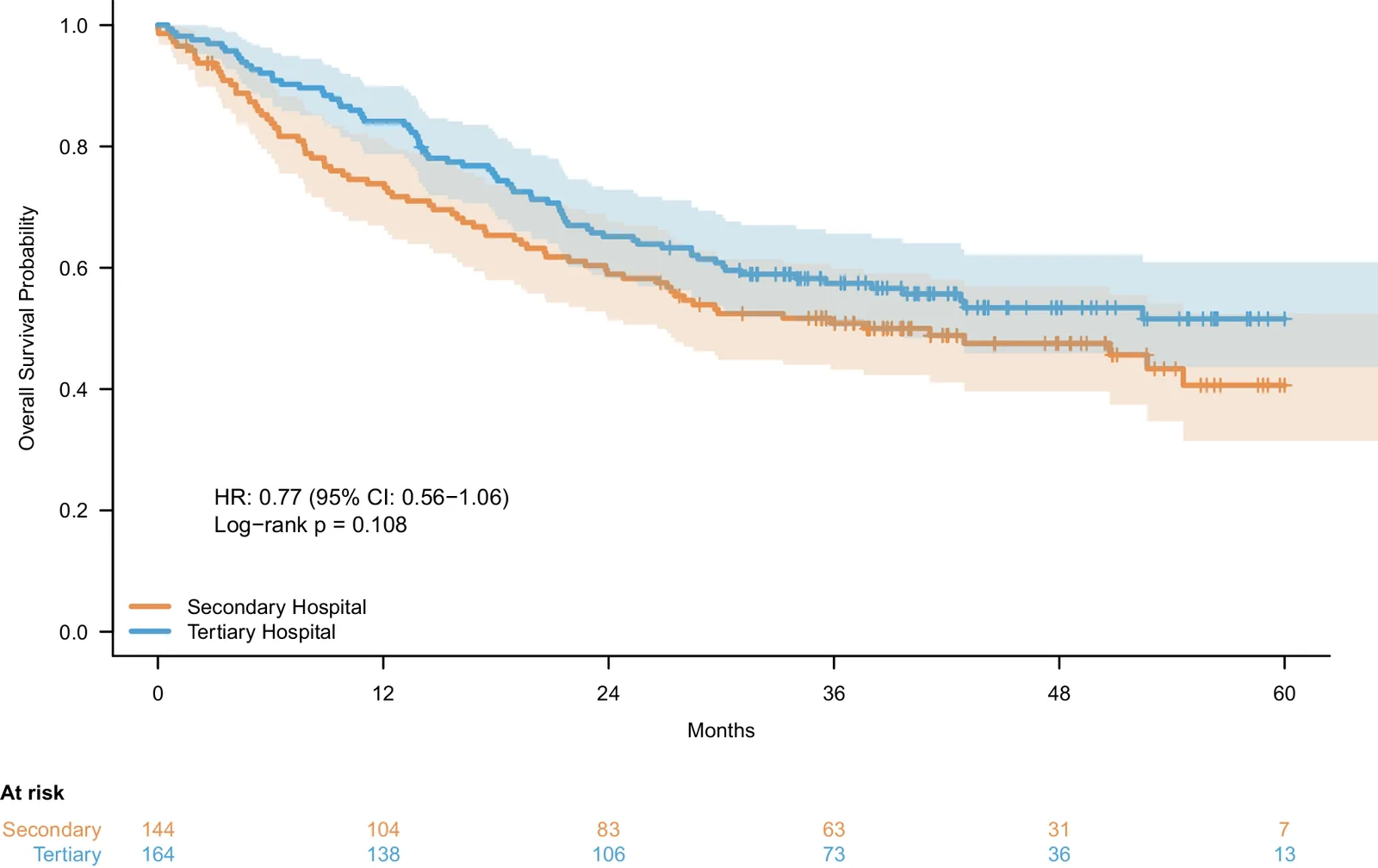
Kaplan-Meier curves for overall survival (OS) stratified by treatment at tertiary versus secondary regional hospitals

**Fig. 4:**
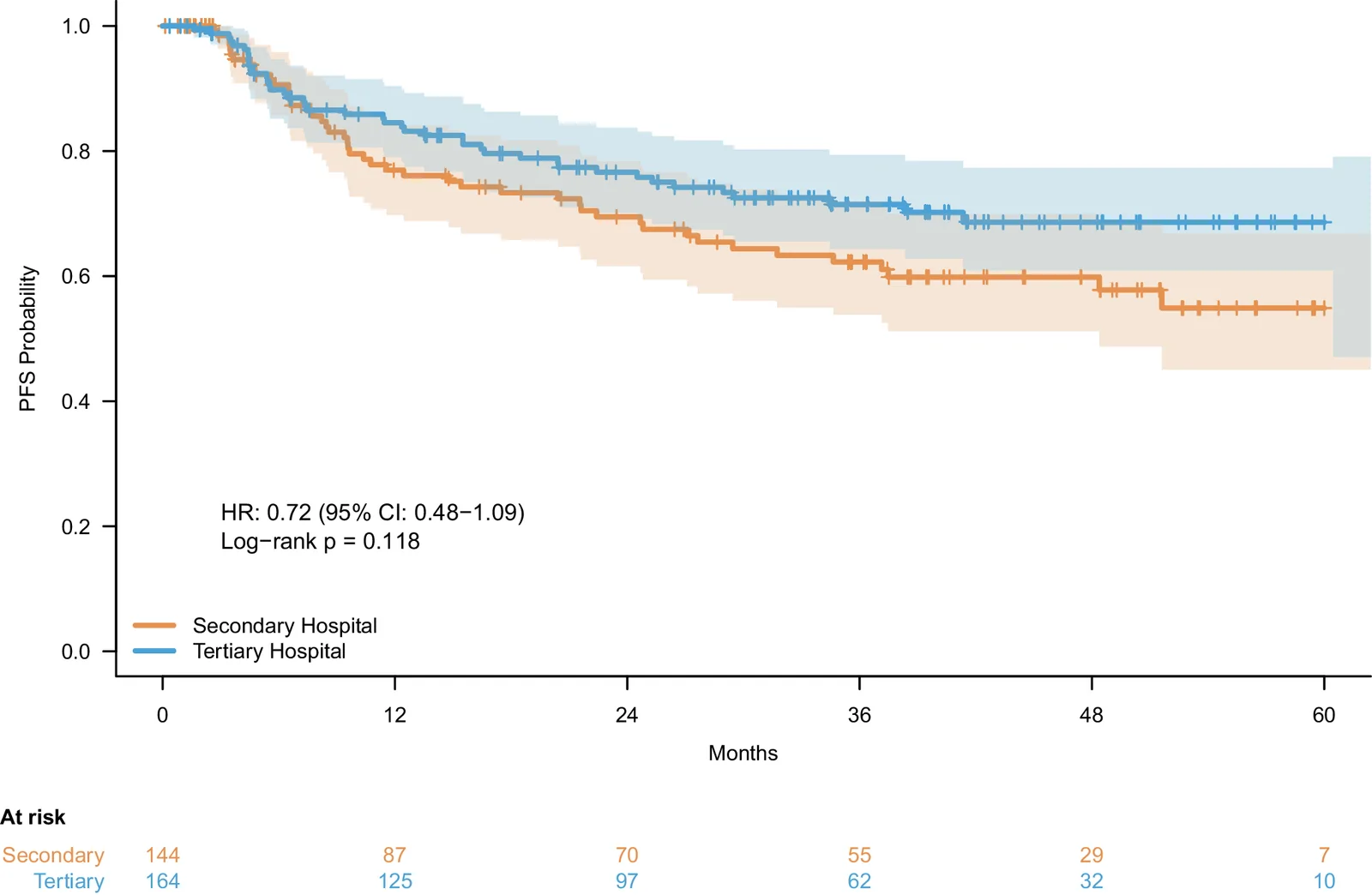
Kaplan-Meier curves for progression-free survival (PFS) stratified by treatment at tertiary versus secondary regional hospitals

**Fig. 5:**
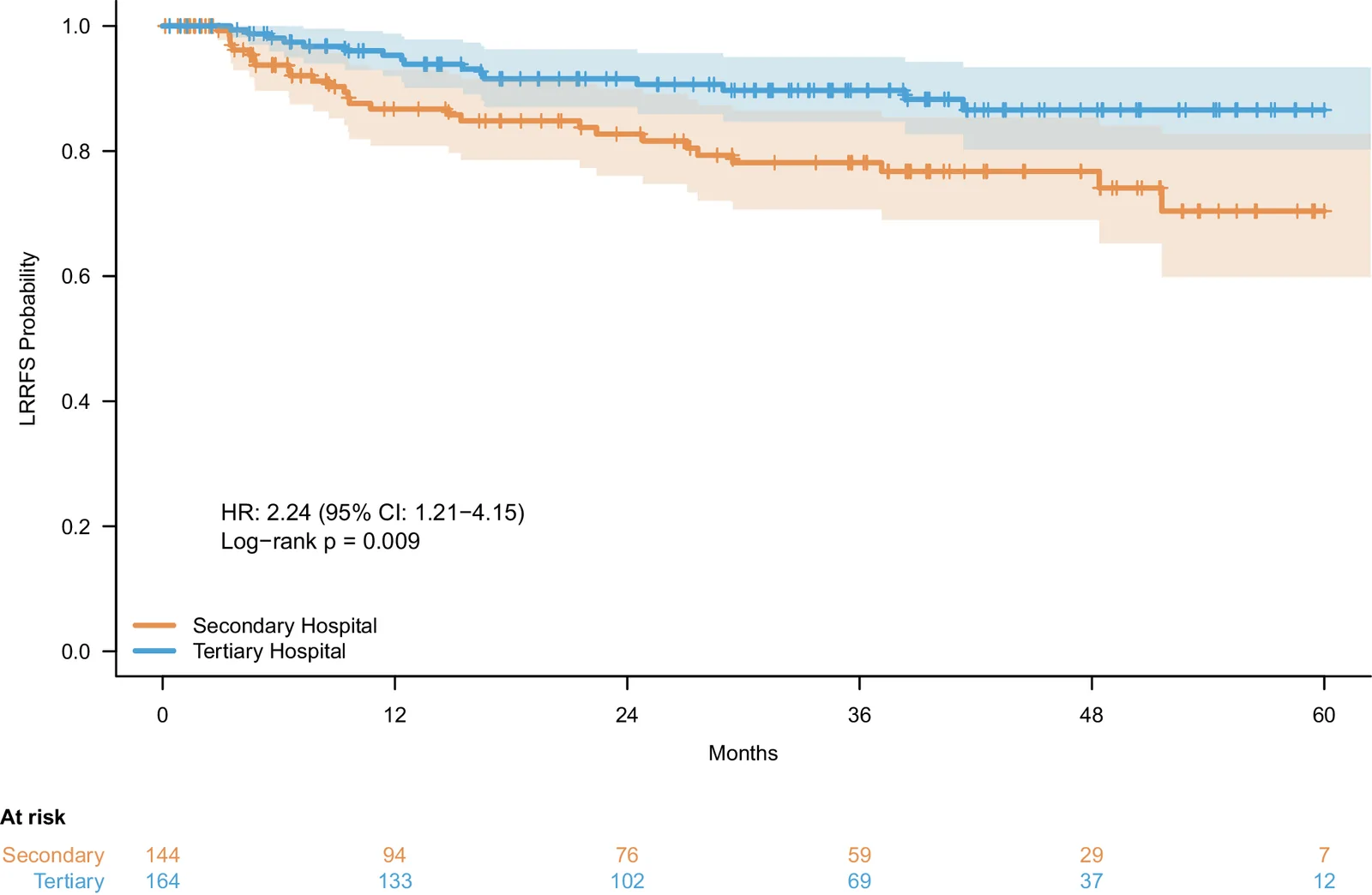
Kaplan-Meier curves for locoregional recurrence-free survival (LRRFS) stratified by treatment at tertiary versus secondary regional hospitals

Median follow-up time was 34 months (IQR 22–43). Kaplan-Meier analyses showed no significant differences in OS (median 35.5 vs 32 months; HR 0.90, 95% CI 0.72–1.12, *p* = 0.34) or PFS (29 vs 21.5 months; HR 0.86, 95% CI 0.69–1.07, *p* = 0.21) between tertiary and secondary regional hospitals (Figs. 2, 3, 4). In contrast, LRRFS was significantly inferior in secondary hospitals (median 25.5 vs 32 months; HR 1.25, 95% CI 1.06–1.48, *p* = 0.009; Fig. 5). Survival endpoints and their interpretation follow established standards in head and neck oncology trials [1, 2].

### Multivariable analysis

In multivariable Cox models, adverse prognostic factors included ECOG > 1 (HR 3.29, 95% CI 1.8–6.0, *p* < 0.001), HPV negativity (HR 2.56, 95% CI 1.4–4.7, *p* = 0.002), and cumulative cisplatin dose < 160 mg/m^2^ (HR 3.12, 95% CI 1.6–6.1, *p* < 0.001). R2 resection status was strongly associated with poor PFS (HR 16.06, 95% CI 3.1–26.8, *p* = 0.022) but not significant for OS. Although UICC stages III and IV differ with respect to T and N stages (as detailed in the revised tables), specific T and N stages were not associated with a different outcome in our multivariable analysis, whereas both remain relevant clinical factors.

### Matched-pair analysis

**Fig. 6:**
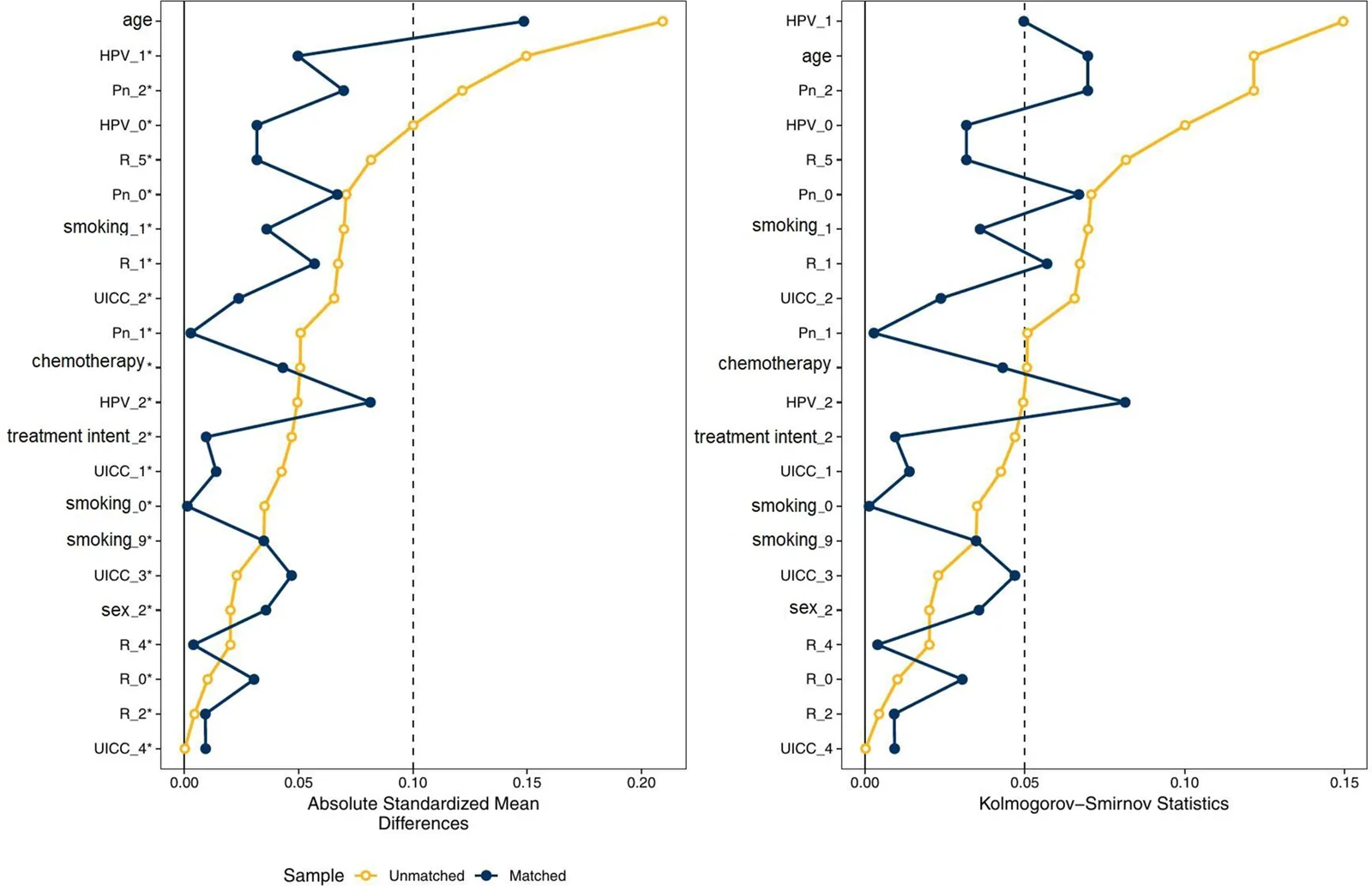
Covariate balance before and after matching. Absolute standardized mean differences (SMD) are shown. Abbreviations: *sex* *1* male, *sex* *2* female; *Smoking_status 0* never smoker, *Smoking_status 1* smoker, *Smoking_status 9* smoking status unknown; *treatment intent 1* definitive radiotherapy, *treatment intent 2* adjuvant radiotherapy; *Chemotherapy 0* without chemotherapy, *Chemotherapy 1* chemotherapy administered; *UICC_1* UICC stage I, *UICC_2* UICC stage II, *UICC_3* UICC stage III, *UICC_4* UICC stage IV; *HPV_0* HPV negative, *HPV_1* HPV positive, *HPV_2* HPV status unknown; *Pn_0* perineural invasion negative, *Pn_1* perineural invasion positive, *Pn_2* perineural invasion unknown; *R_0* R0 resection, *R_1* R1 resection, *R_2* R2 resection, *R4* RX resection status, *R_5* resection status unknown

**Fig. 7:**
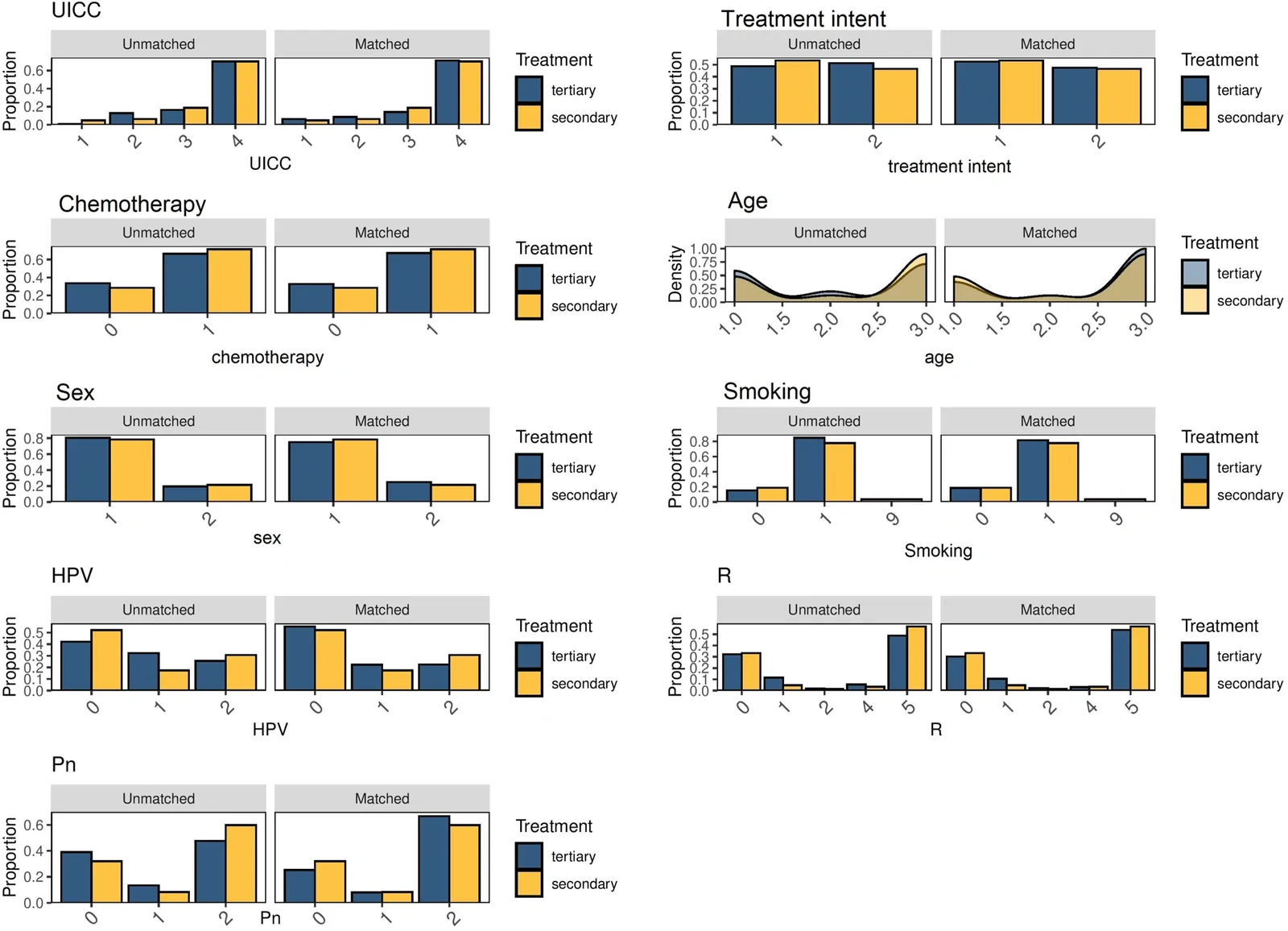
Distribution of key covariates across treatment groups before and after matching. Abbreviations: *sex* *1* male, *sex* *2* female; *Smoking_status 0* never smoker, *Smoking_status 1* smoker, *Smoking_status 9* smoking status unknown; *treatment intent 1* definitive radiotherapy, *treatment intent 2* adjuvant radiotherapy; *Chemotherapy 0* without chemotherapy, *Chemotherapy 1* chemotherapy administered; *UICC_1* UICC stage I, *UICC_2* UICC stage II, *UICC_3* UICC stage III, *UICC_4* UICC stage IV; *HPV_0* HPV negative, *HPV_1* HPV positive, *HPV_2* HPV status unknown; *Pn_0* perineural invasion negative, *Pn_1* perineural invasion positive, *Pn_2* perineural invasion unknown; *R_0* R0 resection, *R_1* R1 resection, *R_2* R2 resection, *R4* RX resection status, *R_5* resection status unknown

After 1:1 optimal pair matching, covariates were well balanced. In the matched cohort, survival outcomes were consistent with the overall analysis: no differences in OS and PFS, but consistently inferior LRRFS in secondary regional hospitals (HR 1.23, 95% CI 1.02–1.48, *p* = 0.03). Covariate balance improved substantially after matching, as illustrated by absolute standardized mean differences (Fig. 6) and by distribution plots of key covariates across treatment groups (Fig. 7). Covariate balance was assessed following established methodological principles [17].

### Sensitivity analyses

Post-hoc power was limited for OS (25%) but moderate for LRRFS (57%). The E‑value for the observed LRRFS difference was 1.81 (lower CI 1.33), indicating that an unmeasured confounder would need to be strongly associated with both exposure and outcome to nullify the observed association [18].

## Discussion

This multicenter cohort study compared treatment patterns and outcomes for patients with pharyngeal and laryngeal squamous cell carcinoma treated with curative-intent radiotherapy or chemoradiotherapy at a tertiary academic radiotherapy center and three secondary hospitals. Overall survival and progression-free survival were comparable across institutions, while inferior LRRFS was observed in secondary hospitals.

Our findings align with reports indicating that institutional case volume alone does not necessarily improve outcome parameters, especially overall survival when contemporary radiotherapy standards are uniformly implemented [13, 14]. Furthermore, a recent retrospective population-based analysis from Belgium demonstrated that higher volume centers (> 20 patients/year) achieved better survival outcomes [27]. Based on this definition, the secondary hospitals presented in our analysis already constitute rather high-volume centers, which likely contributes to the comparable survival outcomes observed.

The observed LRRFS difference was confined to the definitive (non-surgical) treatment cohort, suggesting that biological and clinical factors—particularly the higher proportion of HPV-negative tumors [21–23] and more patients with ECOG > 1 in secondary hospitals—likely contributed to this effect. After multivariable adjustment and matched-pair analysis, a residual LRRFS difference persisted, implying that unmeasured clinical or treatment-related factors may also play a role. The E‑value analysis further supports this interpretation, indicating that an unmeasured confounder would require a risk ratio of at least 1.81 to fully explain the association [18].

Treatment adherence was comparable across hospitals: radiotherapy interruptions were infrequent, and discontinuation rates remained low. Indirect indicators, including universal IMRT/VMAT use, similar prescription doses, and consistent fractionation schemes, support the assumption that secondary hospitals provided radiotherapy of acceptable technical quality, although dosimetric quality assurance was not formally audited [24, 25].

Logistical adherence within our cohort was excellent: the combined rate of unplanned radiotherapy interruptions (≥ 1 day) was 5.5%, and definitive discontinuations (< 30 fractions) were low at 7.8% and comparable between hospitals. This strong logistical compliance contrasts sharply with older randomized trials, in which approximately 16% of patients already exceeded the critical 7‑week overall treatment time, a major protocol violation highly correlated with inferior local control [14]. Homogeneity and efficiency across our hospitals in managing the complex logistical demands of RT are thus clearly demonstrated.

Systemic therapy emerged as a relevant prognostic factor. In our cohort, a cumulative cisplatin dose ≥ 160 mg/m^2^ was associated with improved outcomes. The prognostic relevance of cumulative cisplatin dose is consistent with prior systematic reviews [20]. Whether higher cumulative doses provide additional benefit remains uncertain [26]. Time-to-treatment initiation was shorter at the tertiary hospital, an aspect that may be optimized in secondary hospitals.

This study has several strengths, including real-world multicenter data, comprehensive treatment documentation, and rigorous statistical methodology incorporating matched-pair analysis and sensitivity evaluation. Limitations include the retrospective design, potential selection bias, incomplete HPV testing in a subset of patients, and the absence of formal dosimetric QA audits. Toxicity and quality of life are crucial endpoints in head and neck cancer that may be influenced by treatment volume together with supportive care and central infrastructure. Unfortunately, toxicity and quality of life data were not systematically collected in our dataset, which is a limitation of the present study and therefore cannot be reported. Future prospective studies should include standardized collection of toxicity and functional outcome parameters to better evaluate treatment tolerability and patient quality of life. Follow-up duration may limit interpretation of long-term outcomes, and generalizability is constrained to health systems with comparable structures.

In conclusion, radiotherapy for pharyngeal and laryngeal cancer provided comparable overall and progression-free survival in tertiary and secondary hospitals adhering to contemporary standards. The observed difference in locoregional control appears related to differences in patient and tumor characteristics, with potential contribution from unmeasured factors. These findings highlight the importance of timely treatment initiation, adequate cumulative cisplatin dosing, and continued standardization of radiotherapy procedures across healthcare levels.

## Data Availability

The raw datasets are not publicly available due to patient privacy regulations.
